# Economic impact of muscle injury rate and hamstring strain injuries in professional football clubs. Evidence from LaLiga

**DOI:** 10.1371/journal.pone.0301498

**Published:** 2024-06-13

**Authors:** Laura Nieto Torrejón, Antonio Martínez-Serrano, José M. Villalón, Pedro E. Alcaraz

**Affiliations:** 1 Faculty of Economics and Business, Universidad Católica de Murcia (UCAM), Murcia, Spain; 2 UCAM Research Center for High Performance Sport, Universidad Católica de Murcia (UCAM), Murcia, Spain; 3 Faculty of Sport Sciences, Universidad Católica de Murcia (UCAM), Murcia, Spain; 4 Strength and Conditioning Society (SCS), Murcia, Spain; 5 Club Atlético de Madrid, Madrid, Spain; Wroclaw University of Health and Sport Sciences, POLAND

## Abstract

The aims of this study were: 1) to describe the total muscular injuries, and specifically HSIs, and their corresponding missed matches; 2) to analyse their economic impact; and 3) to estimate the loss of incomes due to TV rights, in first division clubs from LaLiga^TM^ depending on the expected and actual ranking position during the 2018/2019 season. To do that, a cross-sectional study for season 18/19 and for all players of the 20 Spanish professional football clubs was performed. The economic impact of injuries was estimated considering the missed matches and salary cost of all players and the audio-visual income loss was estimated considering the Spanish Royal Decree of Law (RDL 5/2015). The high number of muscular (270) and hamstring injuries (57) implies a high cost for professional first division football clubs, specifically € 365,811 per month for the former and € 47,388 per month for the latter. In addition, reaching a worse than expected position in LaLiga^TM^ ranking involved a loss of 45,2 million € in TV rights incomes. The high cost of muscle injuries in first division teams justifies the need for multidisciplinary teams that are capable of reducing the number of injuries as well as recovery times.

## Introduction

Revenues from TV rights and titles won are the main source of income for European football (soccer) teams, so the increased squad overall performance and player availability are key points [1]. Specifically, in the Spanish Football League (LaLiga^TM^), TV rights range from 23.3% to 81.0% of the total incomes [2], depending on the ranking position at the end of the season. Players contribute by generating income through their sports performance, notoriety, attraction of sponsors, spectators in stadiums, and merchandising sales [3]. In particular, player availability, defined as keeping players injury-free and ready to participate in competition, is extremely important in the current elite team sports scenarios, because it is related to team performance [4], and has been demonstrated to have a close relationship with the number of goals scored [5], and points obtained throughout the season [6], thus determining ranking position [7]. In addition, when a regular professional player is not available due to an injury, he/she is replaced by a less skilled player which often results in quality play decreases, losses of fans or ticketing revenues among others [8].

Therefore, player availability [4] and their management (i.e. injury prevention strategies, return to play and performance, etc.) become a cornerstone towards the achievement of these outcomes [9, 10]. However, despite the overall injury rate showing a downward trend, muscle injury rate has not decreased and neither has its injury burden [11]. Hamstring strain injuries (HSIs) are considered as the most frequent muscle injury in professional soccer [12]. Notably, HSIs have increased during recent seasons, and now constitute 24% of all injuries in men’s professional football [13], causing an average absence time per player of 18.0 days, which increases to 21.5 days when there is a re-injury [14]. This absence not only has a negative impact on the previously mentioned objectives but also entails a considerable financial cost for the clubs.

Previous studies in the Australian Football League stated that the average yearly economic cost of injuries ranged between AUD$ 187,990 to 332,680 [15] and the average financial cost of missed matches due to HSIs increased 71% (AUD$ 148,816 to 245,842) from 2003 to 2012 [16]. This financial loss is especially severe in professional football, where [6] established an approximate loss of £ 45 million per season in the English Premier League (EPL) due to teams’ underachievement (£ 36 million) and the cost of an injured player’s salary (£ 9 million).

Nevertheless, when describing the economic impact of injuries in football in the scientific literature, the average cost of € 500,000 per month for a first team player competing in an UEFA Champions League (UCL) club is frequently used [1]. This value is quoted from a statement by the CEO of a UCL club, thus not based on objectively quantified data. Moreover, there is still limited evidence on the economic impact of muscle injuries and HSIs in professional football, whose data is rarely reported. Therefore, the aims of this study were: 1) to describe the total muscular injuries, and specifically HSIs, and their corresponding missed matches; 2) to analyse their economic impact; and 3) to estimate the loss of incomes due to TV rights, in first division clubs from LaLiga^TM^ depending on the expected and actual ranking position during the 2018/2019 season.

## Methods

### Data collection

A retrospective, cross-sectional study was performed over the 2018–2019 Spanish professional football league (LaLiga^TM^) season. The 20 teams participating in the first division were included in the analysis. Information related to the number and type of injuries and missed matches were collected from clubs’ official web pages, relevant national newspapers such as “Marca^®^” and web pages with publicly available data about the sports industry (http://www.fichajes.com; https://www.futbolred.com/ or http://transfermarket.com/). Only lower limb muscle injuries and HSIs were included for analysis. A muscle injury was defined as a “traumatic or overuse injury to the muscle that led the player to stop participating in training sessions or competitions” [17]. Structural (partial or complete tears) and functional (fatigue-induced muscle damage, hypertonia, or cramps) injuries [17] were considered for analysis whereas contusions or hematomas were excluded. Salaries used to estimate the cost associated with the non-availability of the players were obtained from the previously mentioned sources as well. To estimate the loss of revenue of audio-visual rights derived from the expected position in the ranking, total incomes obtained from audio-visual rights published by LaLiga^TM^ in its annual accounts on the official web page have been taken into account (LaLiga annual accounts).

### Procedures

Firstly, to estimate the economic impact of injuries, we computed the following variables: a) non-availability of players as a consequence of muscle injuries and HSIs; b) associated player cost as a consequence of muscle injuries and HSIs; and c) team overall cost as a consequence of muscle injuries and HSIs.

*Player Non-Availability*. Represents the percentage of LaLiga^TM^ matches for which the player was not available to play due to a muscle injury and a HSI. Player non-availability was calculated for muscular injuries (*NAM*_*p*_) and HSIs (*NAH*_*p*_). To do so, we divided the sum of matches not played by the player (*p*) of a team (t) due to muscular injuries ($\sum {MM}_{t}^{p}$) and HSIs ($\sum {MH}_{t}^{p}$) between the 38 matches. Therefore, *player availability* was the result of (1-*NAM*_*p*_)×100 and (1-*NAH*_*p*_)×100, respectively.


$${NAM}_{p}=\frac{\sum {MM}_{t}^{p}}{38}$$

$${NAH}_{p}=\frac{\sum {MH}_{t}^{p}}{38}$$

*Player Cost*. Annual player cost due to player non-availability caused by muscular injuries (*CM*_*p*_) and HSIs (*CH*_*p*_) was calculated by multiplying *NAM*_*p*_ and *NAH*_*p*_ by the player’s annual salary (*S*_*p*_). In the cases that were not possible to obtain the player’s actual salary, the latest available data or the average salary of the club was used. In 3 cases the last available data was used, and in 12 cases the average salary, which represented 6% and 24% of the total sample of injured players. For the remaining 70% of the players, actual salaries were obtained.


$${CM}_{p}={NAM}_{p}\;x\;S_{p}$$

$${CH}_{p}={NAH}_{p}\;x\;S_{p}$$

*Team Cost*. Team cost for muscular (*TCM*_*t*_) and hamstring (*TCH*_*t*_) injuries refers the total cost per team considering all players and their corresponding non-availability rate. It was calculated as the sum of the costs of each of the players on the team; $\sum {CM}_{t}^{p}$) and $\sum {CH}_{t}^{p}$.


$${TCM}_{t}=\sum {CM}_{t}^{p}$$

$${TCH}_{t}=\sum {CH}_{t}^{p}$$

Secondly, the loss of income due to TV rights is an estimate of the amount of audio-visual rights revenue that a club could have earned if it had achieved the expected position in the final raking. In addition to the amount of revenue it actually earned considering the ranking that the club actually achieved in the season. According to [6], to determine the team’s expected position at the end of the season it is considered the salary threshold established by LaLiga^TM^. LaLiga^TM^ makes a balance between the income and expenses of a club within each season. Therefore, the higher the salary threshold, the better the expected position. In order to estimate the revenues from audio-visual rights that would correspond to each team in both scenarios (i.e., actual and expected rank), the terms of article 5 of RD 5/2015, of April 30^th,^ 2015, on urgent measures in relation to the commercialization of the exploitation rights of audio-visual content of professional soccer competitions was followed. These terms set the criteria for the distribution of revenues among the clubs that participate in the Spanish National League Championship. As mentioned, the volume of audio-visual rights to be distributed was obtained from the audited annual accounts of LaLiga^TM^.

### Statistical analysis

The sample was split into quartiles to present the results. To do this, the 20 teams were sorted according to LaLiga^TM^ actual ranking position and then, divided into four equal groups. The composition of the quartiles was: Q1: FC Barcelona, Atlético de Madrid, Real Madrid, Valencia CF, and Getafe CF; Q2: Sevilla FC, RCD Espanyol de Barcelona, Athletic Club, Real Sociedad, and Real Betis; Q3: Deportivo Alavés, SD Eibar, CD Leganés, Villarreal CF, and Levante UD; Q4: Real Valladolid CF, RC Celta, Girona FC, SD Huesca, and Rayo Vallecano. SPSS^TM^ was used to test the hypothesis about the difference of independent means among these quartiles. T-test for independent samples was used as the categorization of the difference between sample means. The Levene’s test for equality of variances was used. A p<0.05 was the cut-off used for statistical significance. Cohen’s d effect sizes (ES) were also calculated to describe the standardized effects as trivial (<0.2), small (0.2–0.59), moderate (0.6–1.19), large (1.2–1.99), very large (2–4), and near perfect (>4). In addition, a linear regression was performed to model the relationship between missed matches due to muscle injuries or HSIs, and the corresponding actual ranking position.


$$Missed\;matches\;due\;to\;muscular\;injuries=\beta_{0}+\beta_{1}\;x\;Actual\;ranking\;position$$

$$Missed\;matches\;due\;to\;HSI=\beta_{0}+\beta_{1}\;x\;Actual\;ranking\;position$$

## Results

### Lower limb muscle injuries

Injuries, absences, and the cost of lower limb muscle injuries are presented in Table 1. The total number of lower limb muscle injuries during the 2018/2019 season was 270. The sum of injuries resulted in 964 total missed matches. The average availability of players was 87% ± 5%. The average player-salary cost of injuries per club per month was € 365,811 ± € 599,910, specifically, monthly cost ranges from € 15,493 to € 2,328,947.

**Table 1:** Injuries, absences and cost by team.

Team	Muscle injuries	Missed matches due to muscle injuries	HSIs	Missed matches due to HSIs	Global cost
FC Barcelona	18	90	4	13	27.947.368,00
Atlético de Madrid	29	70	4	17	12.784.211,00
Real Madrid	17	76	5	10	18.260.000,00
Valencia CF	22	77	2	2	6.185.789,00
Getafe CF	8	9	0	0	185.921,00
Sevilla FC	15	59	2	5	4.859.737,00
RCD Espanyol	15	48	6	20	1.669.737,00
Athletic Club	10	30	5	15	1.250.526,00
Real Sociedad	19	68	1	2	2.988.421,00
Real Betis	16	63	3	10	2.213.158,00
Deportivo Alavés	5	24	2	4	544.236,00
SD Eibar	11	58	2	4	1.244.369,00
SD Leganés	7	31	2	4	765.392,00
Villarreal FC	8	37	3	18	1.830.526,00
Levante UD	9	25	2	2	501.584,00
Real Valladolid	11	37	1	6	586.017,00
RC Celta	9	42	2	18	1.602.632,00
Girona FC	23	78	3	18	1.694.045,00
SD Huesca	10	23	4	7	327.484,00
Rayo Vallecano	8	19	4	5	353.621,00
Mean	13,5	48,2	2,85	9	4.389.739
Acumulated	270	964	57	180	87.794.774

Table 2, shows the number of injuries, absences and cost by quartiles. The average number of injuries was notably higher in teams ranked in Q1 (18.8), and Q2 (15) in comparison to the injuries of Q3 (8) teams. This difference was statistically significant in both cases (p<0.05; ES = 1.91, and p<0.01; ES = 2.55, respectively).

**Table 2:** Injuries, missed matches, and cost of lower limb muscle injuries.

	Q1	Q2	Q3	Q4	Total
**Total injuries**	94	75	40	61	270
**Injuries per team (mean)**	18.8* (±7.7)	15**¨** (±3.2)	8 (±2.2)	12.2 (±6.1)	54
**Total missed matches**	322	268	175	199	964
**Missed matches per team (mean)**	64.4**¨** (±31.8)	53.6**¨** (±15.1)	35 (±13.9)	39.8 (±23.4)	193
**Total cost per month (€)**	5,446,940	1,081,799	407,176	380,316	7,316,231
**Mean cost per month (€)**	1,089,388* (±895,344)	216,360* (±118,536)	81,435 (±46,734)	76,063⁺ (±56,646)	1,463,246
**Total cost per year (€)**	65,363,289	12,981,579	4,886,107	4,563,799	87,794,774

* Significant differences with Q3 (*p* <0.05); **¨** Significant differences with Q3 (*p* <0.01)

**⁺** Significant difference with Q1 (*p* <0.05)

In addition, teams that were in the first quartile (Q1) had the highest number of missed matches due to muscle injuries. On average, players did not participate in 64.4 ± 32 matches, compared to 39.8 ± 23 missed matches for the teams in the last quartile (Q4). We found a statistically significant difference between the absences of Q1 (p<0.01; ES = 1.20) and Q2 (p<0.01; ES = 1.28) in comparison to Q3, where the mean absences per team reach the minimum missed matches (35). In addition, the linear regression confirms a significant and indirect relationship between missed matches and actual ranking position (*β*_1_ = -0.511; p<0.05); the better the position (from 1 to 20), the greater the number of missed matches.

Regarding player availability, it was similar in all quartiles (Q1 = 85%, Q2 = 87%, Q3 = 85%, and Q4 = 88%).

The total monthly cost of the top five teams was € 5.4 million, representing an annual total cost of € 65.4 million. Regarding the teams placed in the lower quartiles, non-significant differences were found between Q1 and Q2 teams (p = 0.06; ES = 1.37), and significant differences were found between Q1 and Q3 (p<0.05; ES = 1.59), Q1 and Q4 (p<0.05; ES = 1.60), and Q2 and Q3 (p<0.05; ES = 1.50) teams (Table 1).

### Hamstring strain injuries

Injuries, missed matches, and the cost of HSIs are presented in Table 3. The total number of HSIs during the 2018/2019 season was 57. The sum of injuries resulted in 180 total missed matches. The average availability of players was 92%. The average player-salary cost of HSI per club per month was € 47,388 ± € 71,149, specifically, monthly cost ranged from € 0 to € 291,009.

**Table 3:** Injuries, missed matches, and cost of hamstring strain injuries (HSIs).

	Q1	Q2	Q3	Q4	Total
**Total injuries**	15	17	11	14	57
**Injuries per team (mean)**	3 (±2.0)	3.4 (±2.1)	2.2 (±0.4)	2.8 (±1.3)	11.4
**Total missed matches**	42	52	32	54	180
**Missed matches per team (mean)**	8.4 (±7.2)	10.4 (±7.3)	6.4 (±6.5)	10.8⁺ (±6.6)	36
**Total cost per month (€)**	584,430	201,273	102,105	59,957	947,765
**Mean cost per month (€)**	116,886 (±117,520)	40,255 (±25,876)	20,421 (±30,726)	11,991 (±6,662)	189,553
**Total cost per year (€)**	7,013,158	2,415,288	1,225,253	719,480	11,373,179

**⁺** Significant differences with Q1 (*p*<0.05)

The number of HSIs was similar in all quartiles, presenting no statistically significant differences among them. Teams that were in Q2 had the highest number of injuries (17) followed by Q1 (15 injuries). The minimum was Q3 with 11 injuries among its teams.

Teams that suffered the highest number of absences were those who qualified in Q4 (54 absences) whereas the lowest number was in Q3 (32 absences). There was not any statistically significant difference among quartiles, nor in the linear regression.

In terms of monthly cost, the top five teams in the actual ranking showed losses of € 584,430 giving an average cost per team of € 116,886 per month. In the following quartiles of the actual ranking, the cost related to HSIs was significantly reduced, from a total of € 201,273 in the Q2 teams to € 59,957 in the Q4. In terms of annual cost, this type of injury accounted for up to € 7 million in the first quartile of LaLiga^TM^ 2018/2019 qualifiers.

### Comparison between total lower limb muscle injuries and hamstring strain injuries

**Fig 1:**
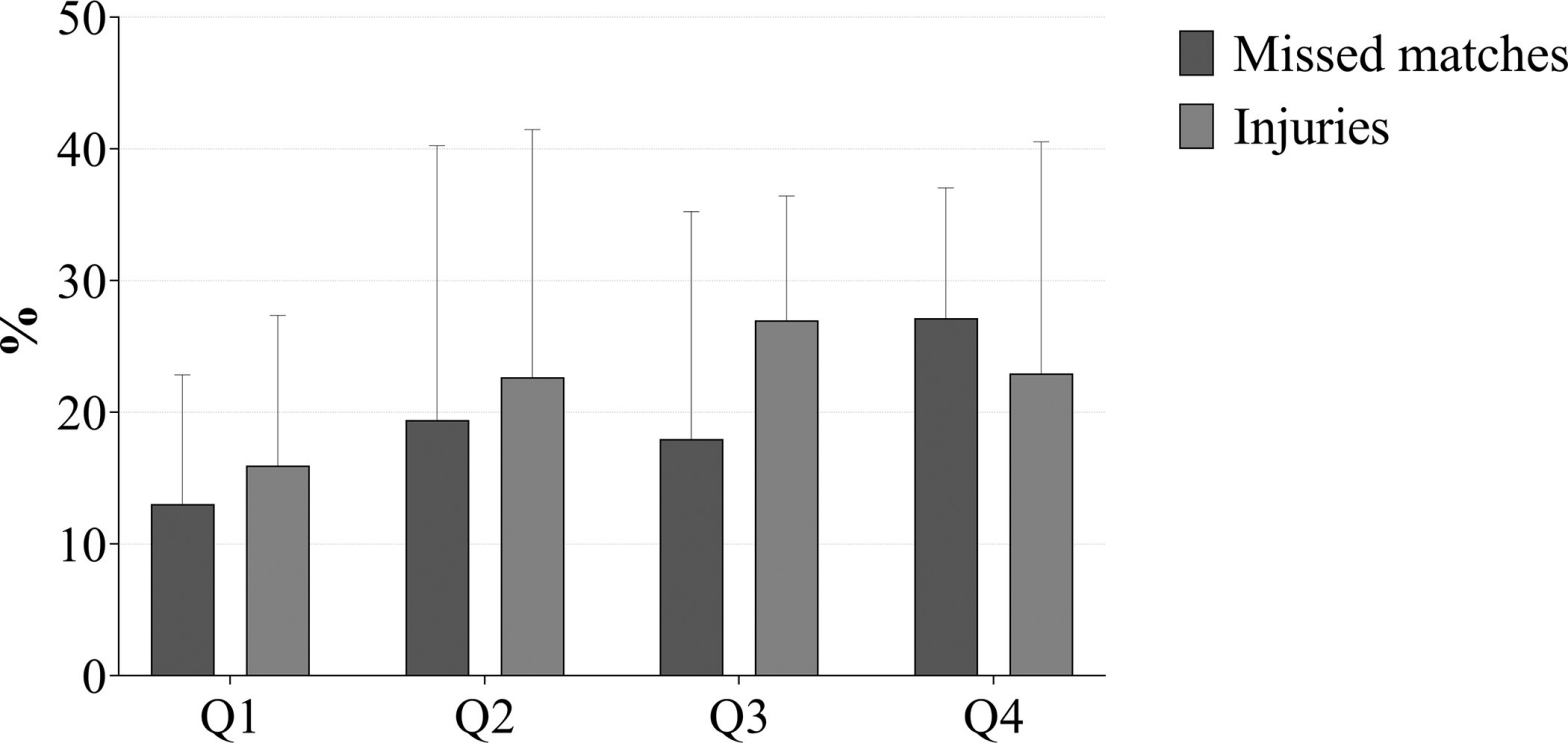
Relative weight of missed matches due to HSIs and number of HSIs over the total number of missed matches and total muscle injuries, respectively.

On average, 21,1% of the team’s muscle injuries were related to hamstrings and accounted for a high proportion of the total cost of muscle injuries (Fig 1). There were 7 teams (35% of the total number of teams) of which more than 26% of the cost due to muscle injuries corresponded to HSIs. Specifically, the relative proportion varied between 26% and 60%.

The proportion of muscle injuries that were classified as HSIs increased as we move down in LaLiga^TM^ actual ranking (Fig 1). While in the Q1 teams, the incidence of HSIs was 16%, the incidence increases to 28% and 23% in the Q3 and Q4, respectively. However, there were no statistically significant differences among quartiles for this ratio.

Similarly, missed matches due to HSIs were higher the worse the actual ranking was, moving from an average absence of 13% of matches in Q1 to 27% in Q4. Only the difference between Q1 and Q4 was statistically significant (p<0.05; ES = 1.71).

### Financial losses from audio-visual rights

In the season 18/19, a total of € 1.4 billion was distributed among first division clubs. According to the Spanish Royal Decree of Law (RDL 5/2015), half of this amount was distributed equally among clubs; 25% was distributed considering their social impact and, the remaining 25% was allocated according to LaLiga^TM^ final ranking. The economic difference between reaching the first or second position was € 6.4 million, between the first or third position was € 12.9 million, and between the first and fourth position was € 19.3 million. Even more remarkable was the difference between the first and last team in the actual ranking with € 52.4 million difference.

A total of 9 football teams were found to be in a lower position than expected, accumulating a loss of income from audio-visual rights of € 45.2 million. S1 Table shows the actual and expected ranking by team.

## Discussion

To the best of the author’s knowledge, this is the first study that presents economic data from the LaLiga^TM^ in terms of the cost of muscle injuries. In addition, it is the first study that presents the economic cost of a professional football league for HSIs. The main findings of this study were that: 1) there was a high economic cost per club due to muscle injuries (€ 365,811 per month and team) and specifically to HSIs (€ 47,388 per month and team), which accounted for 21.1% of the total muscle injuries; 2) player availability was 87%; 3) teams in Q1 of the actual ranking had a higher number of missed matches due to muscle injuries, as well as a higher number of total injuries; 4) the highest number of absences due to HSIs occurred on teams ranked in Q4 and these absences had an inverse relationship with the final ranking position; and 5) placing higher or lower than expected resulted in the teams losing or gaining ~ € 60 million, respectively.

The total cost per month and team in LaLiga^TM^ as a result of muscle injuries was € 365,812, which is lower than the EPL (£ 750,000) [6] but much higher than other “minor” leagues such as the Australian league (AUD$ 23,700 to 41,585 per month and team) [15]. It could be explained by the difference in salary limits between the two leagues and by the incidence of injuries in the teams. LaLiga^TM^ team’s average monthly cost ranges between € 76,063 (Q4) and € 1,089,388 (Q1). The total cost per month of the top five teams was € 5.4 million while in the last five teams was € 380,316. In terms of total cost per year, the accumulated cost was € 65.4 million for Q1 teams, and € 4.5 million for Q4 teams, respectively. Significant differences were found between Q1 and the last two quartile teams (Q3 and Q4). As a result, the total cost of the top teams was 14 times higher than the teams ranked at the bottom. The teams better positioned in the actual ranking showed a higher and significant cost due to injuries because they were also the ones with the highest salaries. This result could be explained by the fact that clubs with higher salary caps have access to better players and have greater financial capacity to retain players who perform at a high level. Thus, the chances of achieving a better position in the expected ranking are multiplied, but the cost of injury to players in these teams is much higher than in clubs with lower salary limits. As an example, taking as a reference the average salary per player, each missed match of the two teams with the highest salary limit would cost € 310,526 (F.C. Barcelona) and € 237,380 (Real Madrid); while in the two teams with the lowest limits (S.D. Huesca and Real Valladolid CF), each missed match would mean approximately, € 16,000 of loss for each club.

Regarding the cost of HSIs, the total cost per month for Q1 teams was € 584,430 and € 59,957 for Q4 teams. This implies an approximated total cost per year of € 7 million for Q1 teams and € 719,480 for Q4, respectively. The average cost per quartile varied between € 11,991 in Q4 and €116,886 in Q1. Although the number of HSIs was similar in all quartiles, teams that suffered the highest number of missed matches were those that qualified in the Q4 (54 missed matches). It must be noted that, on average, 21.1% of the muscle injuries were hamstring injuries. This percentage is in line with the results presented by [13]. In their study, hamstring injuries represent 24% of total injuries in the 21/22 season in professional European clubs that play in European competitions.

The total number of missed matches and muscle injuries were significantly higher in the teams ranked in the top positions. A statistically significant difference was found between the number of injuries of the teams in Q1 and Q2, compared to the number of injuries of the teams in Q3. According to the statistics published by Sports References (https://fbref.com/es/comps/12/2018-2019/Estadisticas-2018-2019-La-Liga), this could be since teams in Q1 accumulated a total of 271 matches (FC Barcelona = 60; Atlético de Madrid = 51; Real Madrid = 55; Valencia CF = 61; and Getafe CF = 44) versus the 206 matches of Q4 teams (SD Huesca = 40; Rayo Vallecano = 40; Girona FC = 44; RC Celta = 40; and Real Valladolid CF = 42). The additional games played in Champions League, Europa League, Copa del Rey, UEFA Supercup, and Spanish Supercopa probably cause a competitive overload in the Q1 players. In fact, a recent systematic review suggests that short-term fixture congestion may increase the match injury incidence [18]. In addition, this competitive overload could be reflected in the increased number of sprints that have been observed season to season in professional soccer [19, 20], as well as an increased neuromuscular fatigue [21]. These factors could increase the volume of neuromuscular load, especially in the posterior chain muscles (e.g., hamstrings). [22] presented a model of the relationship between the number of hamstring injuries and sprint dosage, establishing a “U” shaped relationship, so that an inadequate dosage, both under and over, increased the risk of injury significantly. Our result contrasts with [23] who found that there is an inverse relationship between the playing level and recurrent injury proportions in men´s football. They suggested that professional teams have a larger competitive roster that lets injured players spend more time on rehabilitation pitch with a lesser impact on the team´s performance. Although, a priori, a better expected ranking position implies a larger budget and therefore, a greater rotation of players, this sample demonstrates the opposite. Rotation is not as high as expected in theory. Notably, it can be observed that players with the best performances play almost every game played by the team. As an example, in F.C. Barcelona, Jordi Alba, Ivan Rakitic, and Leo Messi played more than 50 matches during the season, which is 84% of the total matches. In Real Madrid, Karim Benzemá played 51 matches, and Raphael Varane and Luka Modric played more than 40 matches, which represented 85% and 67% of the total matches played by the club, respectively.

On contrary, there were no statistically significant differences between Q3 and Q4 in the total number of missed matches and muscle injuries, it is noteworthy that players in Q4 had higher absences and injuries than players in Q3, which could be explained by two causes. On the one hand, four of the five teams in Q4 did not play in at least two of the three previous seasons in LaLiga^TM^, so the 18/19 season could imply a higher competitive demand for these teams than usual. For example, [24] concluded that being promoted from the second to the first division of Spanish professional football requires players to adapt to greater physical demands and a reduced number of technical actions. In addition, soccer players in a phase of excessive training or intense competition seem to be particularly vulnerable to injury and psychological stress [12]. On the other hand, teams in the Q4 are most likely to leave the league if their results are not as expected. This fact is reflected in the coach changes that usually occur throughout the season to achieve good results that keep the team in the first division. [25] concluded that players reporting the coach as a source of stress are at greater risk of sustaining an overuse injury. In the present study, in four of the five teams assigned to Q4, managerial changes took place in season. This may lead to stress accumulation, fatigue, and its concomitants (i.e., non-functional overexertionor overtraining), and consequently, may increase the risk of injury and illness of the athlete [26]. Therefore, psychology-based interventions should be considered by expert personnel when designing injury prevention programs [27] as well as the player’s position for a better understanding of his/her physical effort during matches [28].

Regarding HSIs, the number of these injuries was similar in all quartiles, presenting no statistically significant differences among them. Teams that suffered the highest number of absences were those who qualified in Q4 (54 absences) whereas the lowest number was in Q3 (32 absences). In a recent study in male professional soccer, pattern analysis revealed 25 sprint-related hamstring injuries (48%) and 27 stretch-related (52%). However, all sprint-related hamstring injuries occurred during linear acceleration or high-speed running [29]. It should be remarked that in professional soccer (EPL) the total distance travelled during a match remained relatively constant, but high-intensity running distance and sprint distance increased by ~30–35% between 2006–07 and 2012–13 [30]. These increases have occurred across all teams regardless of position, for all Tiers the most pronounced increases in physical performance were for explosive metrics such as high intensity running and sprinting [19]. Similarly, [20] observed that the number of efforts performed in high-intensity running increased over eight seasons (2012–2019) in LaLiga^TM^. All this may explain the present results, mainly, because of this increase in the number of actions at very high intensity that causes about 50% of hamstring injuries.

Similarly, missed matches due to HSIs in relation to total muscle injuries were higher the lower the actual ranking was, moving from an average absence of 13% of matches in Q1 to 27% in Q4. Only the difference between Q1 and Q4 was statistically significant and large. In addition to the direct relationship existing between the high volume of sprints and the increased risk of hamstring injury [22] the knowledge about HSI is still limited, therefore a better understanding of its causes, treatments, and rehabilitation is needed [31]. As well, a correct implementation of multidisciplinary preventive programs is necessary to reduce its incidence. A case of success can be seen in the study by [32] where a multicomponent preventive training program was applied, with an individualized approach based on the player’s needs, training load management, physiotherapeutic treatment, and planned communication of all staff. The results showed that the injury rate was 3 times lower during the two intervention seasons than during the previous seasons (p < 0.01); the match injury rate was 2.7 times lower (p < 0.01); and the training rate was 4.3 times lower (p < 0.01). Teams with a higher budget have larger multidisciplinary teams and are managing this injury optimally compared to teams with a smaller budget where the coaching staff is much reduced (data obtained from http://transfermarket.com/). In addition, this could be explained by the greater investment that is being made in clubs competing at the European level, where the technical staff, and especially the material resources for greater control of the load and knowledge of the state of fatigue and recovery of the players is greater, allowing the recovery much faster and lower recurrent injury proportions. These results entail the need to develop specific muscle injury prevention programs and, above all, the importance of multidisciplinary work with highly specialized personnel [33].

Finally, despite the efforts made by LaLiga^TM^ in recent years to reduce the revenue gap between first division clubs, there is a remarkably large difference in revenues among clubs. The 50% of the revenues from audio-visual rights are not distributed equally but according to two indicators: 25% according to the social impact generated by the club and 25% according to its performance. These distribution criteria, approved in RDL 5/2015, are in line with those implemented by other major European leagues such as the English, French, or Italian leagues, where the percentages and distribution indicators are very similar. It is therefore clear that, the distribution of revenues from audio-visual rights tends to depend on the team’s performance, with the players’ availability being of particular importance. Specifically, in the 18/19 season, the loss of audio-visual rights revenue for teams that underperformed was € 45.2 million.

The limitations of the present study are that only a single season was analysed and injuries may fluctuate from one season to another so that in the future it would be interesting to carry out a time-series study as well as to carry out the study for other European leagues. On the other hand, the injury data were obtained from different public databases, so it would be interesting to have access to a database that is contrasted with the clubs themselves. To better estimate the true economic impact of injuries from clubs one should consider all types of injuries and find out the actual salary of those players for whom it is not publicly available. This report also did not consider the financial costs associated with the diagnosis and management of injuries including imaging, therapeutic modalities, surgical interventions, and employment of specialist staff. The impact of player quality unavailable due to injury, injury replacements, competition demands, and squad rotation were not considered in this analysis. As such, it is likely that current analyses without consideration of other factors that may have an impact on injuries and team performance, could lead to misleading inferences.

## Conclusions

During the season 18/19 LaLiga^TM^ first division teams accumulated a total of 270 muscle injuries, 57 of them (21.1%), corresponded to hamstring injuries. This injury incidence represented a total of 964 and 180 missed matches, respectively. A statistically significant difference was found between clubs ranked in Q1 and Q2 compared to Q3 for the number of injuries and missed matches. On the contrary, this pattern did not arise in HSIs. Missed matches due to injuries imply a strong economic cost to the clubs, specifically, muscle injuries present an average monthly cost of € 365,811 per month (total monthly cost of € 7,3 million) and in the case of HSIs, the average monthly cost is about € 47,388 (total monthly cost of € 947,765). Finally, the fact of not reaching the expected position in LaLiga ranking also implies a loss of income for the clubs. Specifically, in the 18/19 season, this loss is estimated at € 45,2 million.

### Practical implications

As practical implications and to reduce costs, the professional clubs must invest in specialized multidisciplinary teams, which include: performance directors, who coordinate the work and increase the quality in communication between the different departments as strength and conditioning, rehabilitation, control of training load and recovery, medical and physiotherapy services, nutrition and psychology. In addition, they must incorporate multi-component work, both in prevention and in returning to competition and performance. These types of actions have been proven effective in significantly reducing the number of injuries and days of absence once it has occurred, especially for the teams that occupy the last positions of the classification where the periods of absence are longer. On the other hand, those responsible for federations and professional leagues must review the number of competitions that are increasing year after year since it may be one of the main causes of the greater number of injuries in players of teams that compete in international competitions. Future research would include a comparison among European leagues and the total injuries suffered by players using a database that is contrasted with the clubs themselves.

## Supporting information

S1 Table Actual and expected ranking of LaLiga^TM^ football clubs. (DOCX)
