# Supplementary material for: Economic impact of muscle injury rate and hamstring strain injuries in professional football clubs. Evidence from LaLiga
Source: PLoS One. 2024 Jun 13;19(6):e0301498. doi: 10.1371/journal.pone.0301498 (PMC11175487; doi:10.1371/journal.pone.0301498)
Supplement: S1 Table — (DOCX) [file pone.0301498.s001.docx]

**Table S1** Actual and expected ranking of LaLiga^TM^ football clubs.

| **Club** | **Expected rank** | **Real Rank** |  | **Club** | **Expected rank** | **Real Rank** |  |
| --- | --- | --- | --- | --- | --- | --- | --- |
| FC Barcelona | 1 | 1 | 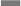 | CD Leganés | 11 | 13 | 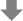 |
| Real Madrid | 2 | 3 | 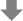 | Levante UD | 12 | 15 | 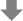 |
| Atlético de Madrid | 3 | 2 | 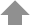 | RC Celta | 13 | 17 | 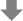 |
| Valencia CF | 4 | 4 | 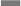 | SD Eibar | 14 | 12 | 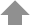 |
| Sevilla CF | 5 | 6 | 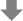 | Getafe CF | 15 | 5 | 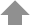 |
| Villarreal CF | 6 | 14 | 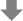 | Deportivo Alavés | 16 | 11 | 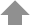 |
| Real Betis | 7 | 10 | 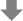 | Girona FC | 17 | 18 | 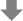 |
| Athletic Club | 8 | 8 | 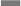 | Rayo Vallecano | 18 | 20 | 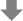 |
| Real Sociedad | 9 | 9 | 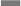 | SD Huesca | 19 | 19 | 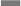 |
| RCD Español | 10 | 7 | 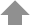 | Real Valladolid | 20 | 16 | 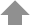 |
